# Incidence and influence on referral of primary stabbing headache in an outpatient headache clinic

**DOI:** 10.1007/s10194-010-0283-3

**Published:** 2011-01-06

**Authors:** A. L. Guerrero, S. Herrero, M. L. Peñas, E. Cortijo, E. Rojo, P. Mulero, R. Fernández

**Affiliations:** Neurology Department, Hospital Clínico Universitario, Avda Ramón y Cajal 3, 47005 Valladolid, Spain

**Keywords:** Primary stabbing headache, Headache clinic, Primary care

## Abstract

Primary stabbing headache (PSH) is a pain, as brief, sharp, jabbing stabs, predominantly felt in the first division of trigeminal nerve. Population studies have shown that PSH is a common headache. However, most people suffer attacks of low frequency or intensity and seldom seek for medical assistance. There are few clinic-based studies of PSH, and its real influence as a primary cause for referral to neurology outpatient offices is to be determined. We aim to investigate the burden of PSH as main complaint in an outpatient headache clinic. We reviewed all patients with PSH (ICHD-II criteria), attended in an outpatient headache clinic in a tertiary hospital during a 2.5-year period (January 2008–June 2010). We considered demographic and nosological characteristics and if PSH was main cause of submission. 36 patients (26 females, 10 males) out of 725 (5%) were diagnosed of PSH. Mean age at onset 34.1 ± 2.9 years (range 10–72). Mean time from onset to diagnosis 68.8 ± 18.3 months. Twenty-four patients fulfilled ICHD-II criteria for other headaches (14 migraine, 6 tension-type headache, 2 hemicrania continua, 1 primary cough headache and 1 primary exertional headache). 77.7% of patients were submitted from primary care. In 14 patients (39%), PSH was main reason for submission, its intensity or frequency in 5 (35.7%) and fear of malignancy in 9 (74.3%). Only two patients of those who associated other headaches were submitted due to PSH. In conclusion, PSH is not an uncommon diagnosis in an outpatient headache office. However, and according to our data, it is not usually the main cause of submission to a headache clinic.

## Introduction

According to the International Classification of Headache Disorders, second edition (ICHD-II) (Table 1) [1], primary stabbing headache (PSH), is a head pain occurring as a single stabs or series of stabs, exclusively or predominantly felt in the distribution of the first division of trigeminal nerve. Stabs are brief, lasting for up to a few seconds and recurring with irregular frequency. To fulfill criteria, there must be no accompanying symptoms or other disorder to attribute pain. Jabbing pains are usually unilateral and unifocal, but sometimes even multifocal and bilateral [2, 3]. Despite mentioned criteria, other locations outside trigeminal region are possible, even in extracephalic region [4, 5].

**Table 1:** Diagnostic criteria of primary stabbing headache in ICHD-II

A	Head pain occurring as a single stab or series of stabs and fulfilling criteria B–D
B	Exclusively or predominantly felt in the distribution of the first division of the trigeminal nerve (orbit, temple and parietal area)
C	Stabs last for up to a few seconds and recur with irregular frequency ranging from one to many per day
D	No accompanying symptoms
E	Not attributed to another disorder

Population studies have shown that PSH is a common headache [6]. However, most people suffer attacks of low frequency or intensity and seldom seek for medical assistance. There are few clinic-based studies of PSH, and its real influence as a primary cause for referral to general neurological or headache outpatient offices is to be determined. We aim to investigate the burden of PSH as main complaint in an outpatient headache clinic.

## Methods

We prospectively reviewed all patients with PSH diagnosed in accordance with ICHD-II criteria, attended in a headache outpatient office in a tertiary hospital during a 2.5-year period (January 2008–June 2010). In each patient, we considered demographic and nosological characteristics and if PSH was the main cause of submission to our office. In our Public Health Network, SACYL (Autonomous Community of Castilla y Leon, Central Spain), patients are referred to neurology outpatient offices by general practitioners who act as gatekeeper, and they cannot self-refer to neurology clinic. Besides, some patients were submitted from general neurology offices or other specialities, mainly neurosurgery.

## Results

A total of 725 patients were attended in our headache clinic during inclusion period. 36 (5%) of them (26 females, 10 males) were diagnosed of PSH accordingly to ICHD-II criteria. Mean age at onset was 34.1 ± 2.9 years (range 10–72) and did not differ between men and women. Mean time from onset of symptoms to diagnosis was 68.8 ± 18.3 months.

Regarding headache profile, nature of pain was stabbing in all patients. Jabs were bilateral in 28 cases (78%) and, when unilateral, equally distributed between right and left side (*n* *=* 4, 11%). 16 patients (44%) felt pain paroxysms in any region of the head. When jabs were restricted to a fixed area, they were more frequently localized in frontal area (*n* *=* 12, 33%), followed by parietal (*n* *=* 3, 8%), occipital (*n* *=* 3, 8%) and temporal (*n* *=* 2, 5%).

Mean intensity of jabs, measured according to a visual analogical scale (0 no pain, 10 the worst pain imaginable) was 6.1 ± 0.4 (range 3–8). 23 patients (64%) described duration of a single jab under 5 s and 21 (58%) reported more than a jab per day. Twenty-four patients (67%) fulfilled ICHD-II criteria for other headaches. 14 of them (12 females, 2 males) migraine with or without area, 6 (5 females, 1 male) tension type headache, 2 hemicrania continua, 1 primary cough headache and 1 primary exertional headache.

28 (77.7%) patients were referred to our headache clinic from primary care, 2 (5.6%) from other neurology offices, and 6 (16.7%) from other specialities, mainly neurosurgery. In only 14 patients (39%), PSH was the main reason for headache clinic consultation and, among them, submission was due to frequency or intensity of stabs in 5 patients and to fear of malignancy in 9 cases. Only 2 patients among 24, who associated other headaches, were referred due to PSH.

Regarding therapy, 10 patients received indomethacin for PSH. Mean dose of indomethacin was 130 ± 35 mg and 20% patients had complete remission and 70% partial response. In 13 patients, a preventative for the comorbid headache (6 topiramate, 2 amitriptyline, 2 beta-blockers and 2 indomethacin) was prescribed; 30% of them presented a complete remission and 70% a partial response.

## Discussion

Primary stabbing headache is a prevalent, but probably quite unknown disorder, classified in ICHD-II as a primary headache syndrome, with a female preponderance and onset at middle age, as is found in our series [6–8]. Consistent with the previous reports [1, 7, 9], our study shows that most attacks last 5 s or less. In our series, most patients present more than one stab per day. Accompanying symptoms during attacks as allodynia, nausea/vomiting or photophobia/phonophobia have been reported, mainly when paroxysms are more intense [7, 10].

Although fronto-ocular and temporal regions are the most frequently affected [2, 6], some reports have shown that PSH can occur outside the trigeminal region, mainly occipital or nuchal, and even in the extracephalic region [4, 5]. The attacks frequently change location from one area to the next [4, 7, 9].

Regarding prevalence studies, existence of ultrashort head pain paroxysms has been known for decades; initial population-based studies showed an estimated prevalence of around 2% [3, 11, 12]. However, as paroxysms are usually associated with other headaches, it may be difficult to estimate PSH real prevalence because the associated headaches would receive more attention [3].

The large-scale study of headache epidemiology in Vaga, Norway, carried out several years prior to ICHD criteria, describes a prevalence of jabs suggestive of PSH of 35% [4]. Most of the jabs described by Vaga parishioners were mild, short-lasting and single; only 5% of patients experienced more than five attacks per day [3, 11]. Long-lasting jabs (<1% of described jabs in Vaga study) seem to be associated with migraine [10].

Most people have attacks of low frequency, so perhaps they seldom visit the doctor [3]. Patients admitted in outpatient clinic might, so, be different from those surveyed in the community, but there are few large-scale clinic-based studies of PSH [2, 7].

Recently, two studies registering PSH patients according to ICHD-II criteria in tertiary headache clinic have found a quiet comparable frequency of this headache of 13% [7, 8]. In these two studies is unclear whether patients contact Hospital only due to PSH; besides, these patients commonly complain of other primary headaches, usually migraines, probably the main cause of consultation. Mean reasons of consultation described are severity of pain or fear of stroke or malignancy.

Pharmacologic treatment is rarely necessary and usually is prescribed due to a comorbid headache disorder as occurs in our series. When the frequency of attacks is high, indomethacin is the medication of choice [1, 8, 13]. Other possibilities are melatonin, gabapentin and celecoxib and even the botulinum neurotoxin with different results [14].

## Conclusion

Although PSH is not uncommon in population or headache clinic-based studies, it may be, according to our results, a quite infrequent diagnosis in a headache outpatient office, especially when considering primary cause of admitting.

Information about this treatable entity should be provided to population and/or primary care physicians.
